# Inductively Coupled Nonthermal Plasma Synthesis of Size-Controlled γ-Al_2_O_3_ Nanocrystals

**DOI:** 10.3390/nano13101627

**Published:** 2023-05-12

**Authors:** Zichang Xiong, Himashi P. Andaraarachchi, Jacob T. Held, Rick W. Dorn, Yong-Jin Jeong, Aaron Rossini, Uwe R. Kortshagen

**Affiliations:** 1 Department of Mechanical Engineering, University of Minnesota, 111 Church Street SE, Minneapolis, MN 55455, USA; xion1832@umn.edu (Z.X.); handaraa@umn.edu (H.P.A.); yjjeong@ut.ac.kr (Y.-J.J.); 2 Chemical Engineering and Materials Science Department, University of Minnesota, Minneapolis, MN 55455, USA; jaheld@ethz.ch; 3 Ames National Laboratory, United States Department of Energy, Department of Chemistry, Iowa State University, Ames, IA 50011, USA; rwdorn@iastate.edu (R.W.D.); arossini@iastate.edu (A.R.)

**Keywords:** gamma alumina, nonthermal plasma, size-control

## Abstract

Gamma alumina (γ-Al_2_O_3_) is widely used as a catalyst and catalytic support due to its high specific surface area and porosity. However, synthesis of γ-Al_2_O_3_ nanocrystals is often a complicated process requiring high temperatures or additional post-synthetic steps. Here, we report a single-step synthesis of size-controlled and monodisperse, facetted γ-Al_2_O_3_ nanocrystals in an inductively coupled nonthermal plasma reactor using trimethylaluminum and oxygen as precursors. Under optimized conditions, we observed phase-pure, cuboctahedral γ-Al_2_O_3_ nanocrystals with defined surface facets. Nuclear magnetic resonance studies revealed that nanocrystal surfaces are populated with AlO_6_, AlO_5_ and AlO_4_ units with clusters of hydroxyl groups. Nanocrystal size tuning was achieved by varying the total reactor pressure yielding particles as small as 3.5 nm, below the predicted thermodynamic stability limit for γ-Al_2_O_3_.

## 1. Introduction

Alumina, Al_2_O_3_, is extensively used for a wide range of applications because of its superior chemical, mechanical, and thermal properties [1,2]. Among the crystalline Al_2_O_3_ polymorphs, gamma alumina (γ-Al_2_O_3_) has attracted significant attention as a catalyst and a catalytic support [3,4,5,6] nanomaterial in heterogeneous catalysis and petroleum refining processes [7,8,9,10] due to its high specific surface area [3,4,5], high porosity [3], and excellent thermal stability [4,9].

Typically, gibbsite (γ-Al(OH)_3_) and boehmite (γ-AlOOH) are used as precursors to synthesize nanostructured γ-Al_2_O_3_ particles [11,12]. Various methods have been developed to synthesize γ-Al_2_O_3_ nanocrystals including precipitation [3,13,14,15,16,17,18], sol-gel synthesis [4,19,20,21,22], combustion [23], hydrolysis [24,25], and high-pressure compaction [26]. It was shown that the final structure, morphology, and properties of γ-Al_2_O_3_ nanocrystals largely depend on the synthetic routes and experimental parameters. However, most of these methods require high-reaction temperatures, long reaction times (~35 h–6 days), and additional calcination steps.

The detailed structure of γ-Al_2_O_3_ still remains unclear owing to the structural complexity and the notorious presence of mixed alumina polymorphs during synthesis [27,28,29]. Many properties and surface characteristics of γ-Al_2_O_3_ are still actively debated [29,30]. Nanostructured γ-Al_2_O_3_ often results in cube octahedral or octahedral morphology demonstrating distinct surface facets [7,31,32]. These surface facets directly influence the anchoring ability, dispersion, and surface interaction of metal catalyst atoms and their catalytic behaviour [7,31]. Reported synthetic methods that utilize high-temperature conditions often produce polydisperse nanocrystal aggregates with non-reproducible facet orientations, which hamper their catalytic activity.

Here, we report a rapid, single-step, low temperature synthesis of phase-pure facetted γ-Al_2_O_3_ nanocrystals using a flow-through nonthermal inductively coupled plasma (ICP) reactor. The nonthermal plasma approach has emerged as a competitive technique in producing a variety of nanoparticles with high-purity and narrow size distributions [33,34,35]. One of the major advantages of nanocrystals synthesis in plasma is the unipolar electrical charging of particles that reduces or eliminates the agglomeration of nanoparticles [33,34]. Exploiting this unique feature, we synthesized γ-Al_2_O_3_ nanocrystals with defined surface planes using a low-pressure nonthermal ICP reactor utilizing trimethylaluminum and oxygen as precursors. Cendejas et al., recently studied the crystallization of Al_2_O_3_ nanoparticles in a nonthermal capacitively coupled plasma (CCP) reactor and achieved the γ-Al_2_O_3_ phase, but their study did not demonstrate control of the nanocrystal size and lacked detailed microstructural characterization [36]. The plasma density of an ICP used in this study is usually more than one order of magnitude higher than that of a CCP [37]. We demonstrate, here, that this causes effective crystallization of the γ-Al_2_O_3_ phase [38,39]. We also performed detailed microstructural characterizations of the synthesized γ-Al_2_O_3_ nanocrystals, which revealed cuboctahedra morphology with (111) stepped facets. Furthermore, size tuning of γ-Al_2_O_3_ nanocrystals was achieved here by varying the total reactor pressure.

## 2. Methods

### 2.1. γ-Al_2_O_3_ Nanocrystal Synthesis

**Figure 1:**
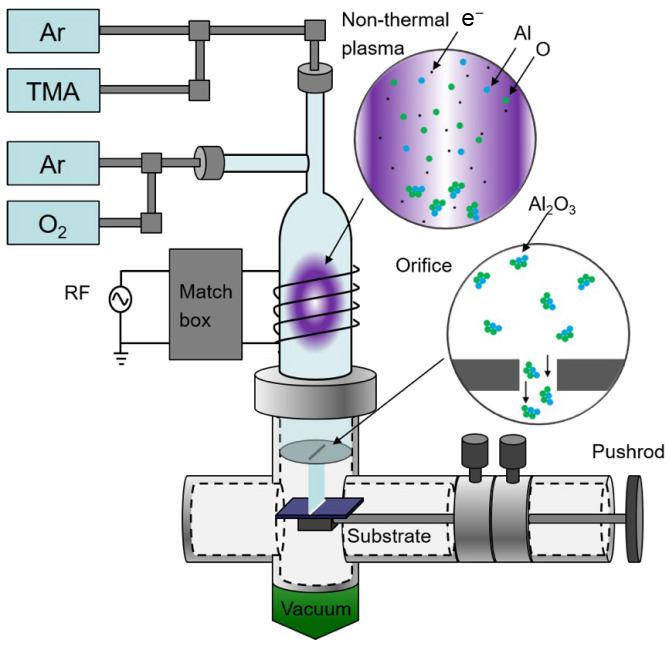
Schematic diagram of the nonthermal ICP setup for the synthesis of γ–Al_2_O_3_ nanocrystals.

γ-Al_2_O_3_ nanocrystals were synthesized using a flow-through, low-pressure, nonthermal ICP reactor, Figure 1, which is similar to a previously published report [40]. A simplified schematic of this system is shown in Figure 1. Briefly, a low-pressure discharge was generated by the application of 120 W radio frequency (13.56 MHz) power to an induction copper coil with three turns, approximately 2.5 cm in length and 2.5 cm in diameter, wrapped around a quartz tube with an outer diameter of 2.5 cm. The RF power was generated by a 13.56 MHz RF power supply (AG 0313, T&C Power Conversion, Rochester, NY, USA) and applied through an impedance matching network (Model HFT1500, Vectronics, Starkville, MS, USA). Trimethylaluminum (TMA, 97%, Sigma Aldrich, St. Louis, MO, USA) vapor, carried by an argon flow, entered through the top inlet mixed with a diluted oxygen/argon flow that entered from the side arm tube before the plasma discharge. In a typical recipe, the TMA volumetric flow rate was about 0.2 standard cubic centimetres per minute (sccm) carried by 6 sccm of argon flowing through the TMA bubbler, in which pressure was stable around 350 Torr. The oxygen volumetric flow rate and the associated argon volumetric flow rate were 2.5 sccm and 60 sccm, respectively. γ-Al_2_O_3_ nanocrystals were collected on silicon wafer substrates by inertial impaction [41] using an orifice with a 0.25 × 8 mm rectangular opening. The total reactor pressure was maintained at approximately 3.8 Torr for the typical recipe. The collection rate of γ-Al_2_O_3_ nanocrystals was 18 mg/h. Deposited nanocrystals were stored under ambient conditions. Size tuning experiments were carried out by varying the orifice diameter, leading to a change in reactor pressure. While the above-described reaction conditions were kept the same for 0.25 mm and 0.5 mm orifice diameters, 15 sccm of Ar flow with TMA and 5 sccm of O_2_ were used for an orifice of 1 mm diameter. The 0.5 mm and 1 mm orifice diameters resulted in total reactor pressures of 2.5 and 1.3 Torr, respectively.

### 2.2. X-ray Diffraction (XRD)

XRD was performed using a Bruker D8 Discover 2D X-ray diffractometer (Bruker, Billerica, MA, USA) equipped with a Co Kα (λ = 1.79 Å) radiation point source in Bragg–Brentano configuration. The diffraction patterns were collected at 25 °C with a step size of 0.01° per step and a retention time of 5 s per increment. Nanocrystals were directly deposited onto Si wafers for XRD analysis. The XRD patterns were converted to Cu source (λ = 1.54 Å) for data analysis. Data analysis was performed using the Material Data Incorporated Jade 8.0 software package.

### 2.3. Fourier-Transform Infrared (FT-IR) Spectroscopy

FT-IR spectroscopy was performed on a Bruker Alpha spectrometer (Bruker, Billerica, MA, USA) using the attenuated total reflection (ATR) module in a nitrogen filled glovebox. The as synthesized nanocrystals were dissolved in methanol, drop cast onto the ATR crystal, and 20 scans were taken for each measurement at 2 cm^−1^ resolution.

### 2.4. X-ray Photoelectron Spectroscopy (XPS)

XPS was performed with a PHI Versa Probe III XPS and UPS system (Physical Electronics, Chanhassen, MN, USA). Samples were directly deposited onto Si wafers for XPS analysis. The binding energy of C 1 s at 284.6 eV was used as a reference. A 55 eV bandpass energy was used to collect high-resolution scans. Peaks were fitted using PHI’s Multipak software v9. XPS survey scans were taken at a bandpass energy of 280 eV. The atomic percentages were calculated using Multipak software v9.

### 2.5. Transmission Electron Microscopy (TEM)

High-angle annular dark-field scanning transmission microscopy (HAADF-STEM) images were collected using an aberration-corrected FEI Titan G2 60-300 (FEI, Hillsboro, OR, USA) operated at 300 kV with a 25 mrad semi-convergence angle. Transmission electron microscopy (TEM) images were collected using a Thermo Scientific Talos F200X (Thermo Scientific, Waltham, MA, USA) operated at 200 kV. Samples were directly deposited onto lacy/thin double carbon-coated TEM grids. Nanocrystal dimensions were measured using ImageJ. A minimum of 300 particles were counted for every size distribution and the nanoparticle dimensions were reported with geometric standard deviations.

### 2.6. Solid-State Nuclear Magnetic Resonance (NMR) Spectroscopy

Solid-State NMR spectroscopy was performed at the National High Magnetic Field Laboratory in Tallahassee, FL, USA, on a 19.6 T (ν_0_(^1^H) = 833 MHz) magnet equipped with a Bruker NEO console (Bruker, Billerica, MA, USA) and a 3.2 mm Low-E HX magic-angle spinning (MAS) NMR probe. The magnet field strength was referenced to the ^17^O chemical shift of tap water [δ_iso_(^17^O) = 2.825 ppm]. ^1^H and ^27^Al shifts were indirectly referenced to 1% TMS in CDCl3 and aqueous Al(NO_3_)_3_ solution, respectively, using the previously published relative NMR frequencies [42]. The as synthesized γ-Al_2_O_3_ nanocrystals were packed into the appropriate sized NMR rotor in a N_2_ filled glove-box. All NMR spectra were processed in Bruker Topspin 4.0.7. The ^1^H π/2 and π pulse lengths were 5 and 10 μs in duration, respectively, corresponding to a 50 kHz radio frequency. ^27^Al central-transition (CT) selective π/2 and π pulse lengths were 5 and 10 μs in duration, respectively, corresponding to a 16.7 kHz RF field and 50 kHz CT nutation frequency. ^1^H and ^27^Al longitudinal relaxation constants (*T*_1_) were measured using a saturation recovery experiment. A quantitative single-pulse ^27^Al NMR spectrum was recorded with a 30 s recycle delay [ca. 10–30 × T_1_(^27^Al)] and a 10° tip-angle excitation pulse. A 2D ^27^Al → ^1^H Dipolar-mediated Refocused Insensitive Nuclei Enhanced by a Polarization Transfer (D-RINEPT) 2D correlation NMR spectrum was recorded with previously described NMR pulse sequences [43,44], and the symmetry-based $\mathrm{SR}4_{1}^{2}$ heteronuclear dipolar-recoupling sequence [45] was applied to the ^1^H spins. A 2D ^1^H dipolar double quantum-single quantum (DQ-SQ) homonuclear correlation NMR spectrum was recorded with the previously described back-to-back (BABA) NMR pulse sequence [46,47]. 2D ^27^Al dipolar DQ-SQ homonuclear correlation NMR spectra were recorded with $\mathrm{BR}2_{2}^{1}\;$ homonuclear dipolar recoupling and the previously described pulse sequences [48]. A CT (central transition) selective π pulse was applied during the indirect dimension (t_1_) evolution period to ensure only DQ coherence between two ^27^Al spins in the CT spin states were observed [49]. Rotor-assisted population transfer (RAPT) was applied to ±400 kHz off-resonance before all ^27^Al → ^1^H D-RINEPT and ^27^Al DQ-SQ NMR experiments to enhance the ^27^Al CT NMR signals [50,51]. Small phase incremental alternation with 64 step (SPINAL-64) [52] heteronuclear decoupling with a 50 kHz ^1^H RF field was applied during the detection of ^27^Al NMR signals.

## 3. Results and Discussion

Freestanding γ-Al_2_O_3_ nanocrystals were synthesized using the flow-through nonthermal plasma process discussed above. Even though nucleophilic molecular oxygen is highly reactive towards the electron deficient TMA under ambient conditions, it is found to be rather inert or unreactive with many organometallic precursors including TMA at low-pressure and low-temperature conditions [53,54]. Thus, the pre-mixing of TMA with molecular oxygen before the plasma region did not lead to a reaction of the gas before entering the plasma. The detailed chemistry occurring in the plasma region is poorly understood, but according to the previous reports, oxidation of TMA will proceed through initial decomposition [53], which is followed by the reaction with atomic oxygen. In the plasma region, TMA is expected to rapidly decompose to produce reactive intermediates such as metal atoms (Al) released by electron impact or partially decomposed fragments generated by radical abstraction reactions. These reactive intermediates will react with atomic oxygen to form fully oxidized γ-Al_2_O_3_ nanocrystals. Nucleation and growth of these nanocrystals are believed to follow the typical nanoparticle growth in plasmas, where the growth proceeds through the nucleation of clusters that rapidly coagulate to form nanoparticles [55]. Crystallization is induced by the heat generated by energetic surface reactions such as electron-ion recombination and surface chemical reactions [36,39]. Due to the higher plasma density of the ICP, and thus more intensive nanoparticle heating, the nominal power reported here (120 W) is around 2.5 times lower than the required nominal power (300 W) [36] for full crystalline nanoparticles using a CCP.

**Figure 2:**
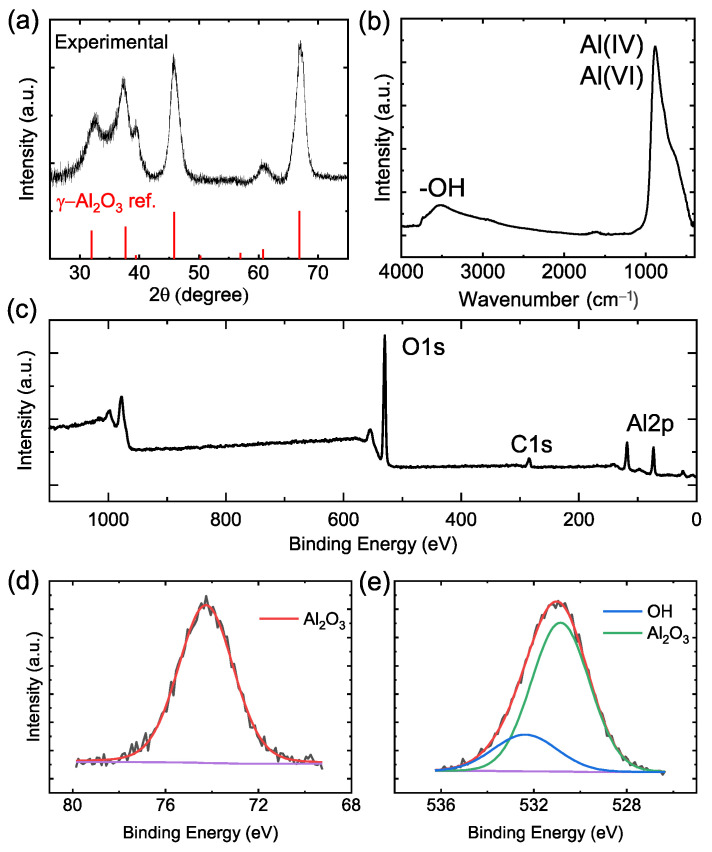
(**a**) XRD patterns of experimental (black) and reference (red) γ-Al_2_O_3_ nanocrystals (PDF number-29-1486). (**b**) FT-IR spectrum, (**c**) XPS survey scan, and (**d**,**e**) high-resolution Al 2p and O 1s spectra, respectively. Purple lines indicate background spectra.

As synthesized γ-Al_2_O_3_ nanocrystals were structurally characterized through the means of XRD, TEM, and FT-IR, XPS, and solid-state NMR spectroscopies. These materials often adopt a defect spinel structure with oxygen atoms forming a face centered cubic structure, and Al cations occupying the interstitial tetrahedral and octahedral sites [12,30,56,57]. Figure 2a shows the theoretical and experimental powder-XRD patterns of γ-Al_2_O_3_ nanocrystals. The experimental pattern exhibits six reflections at 32.0°, 37.9°, 39.9°, 46.2°, 61.2°, and 66.8°, which correspond to the standard γ-Al_2_O_3_ crystal planes of 220, 331, 222, 400, 511, and 440, respectively (JCPDS No. 29-1486).

FT-IR and XPS were performed to evaluate the surface composition of the plasma synthesized γ-Al_2_O_3_ nanocrystals. As shown in Figure 2b, the absorption band ranging from 430–890 cm^−1^ can be attributed to the characteristic γ-Al_2_O_3_ stretching modes of four-fold (AlO_4_) and six-fold coordinated (AlO_6_) Al sites [58,59,60]. While peaks at ~890 cm^−1^ and the shoulder at ~760 cm^−1^ can be assigned to the Al-O stretching modes of AlO_4_, the shoulder at ~620 cm^−1^ relates to the Al-O stretching modes of AlO_6_. The broad absorption band at ~3570 cm^−1^ corresponds to the surface-bound and free –OH groups. In Figure 2c, XPS survey of these nanocrystals revealed three main peaks corresponding to the Al 2 p, C 1 s, and O 1s at 74, 285, and 530 eV lines, respectively. The atomic percentage of C was around 12%, which may originate from the methyl groups in TMA or partly due to the contamination in air as samples were briefly exposed to the air during transfer. A marginal C contamination is commonly observed when TMA is used due to the strong Al-C bonds [61,62]. The high-resolution XPS spectrum of Al showed a single peak at 74.2 eV, which corresponds to Al-O bonding in Al_2_O_3_ (Figure 2d) [63,64]. The O 1s peak at 530.5 eV can be deconvoluted to two individual peaks, indicating –OH and Al-O surface species (Figure 2e) [63,64,65,66]. The observed XPS features are consistent with the observed absorption peaks in the FT-IR spectrum, as well as the solid-state NMR spectra discussed below.

**Figure 3:**
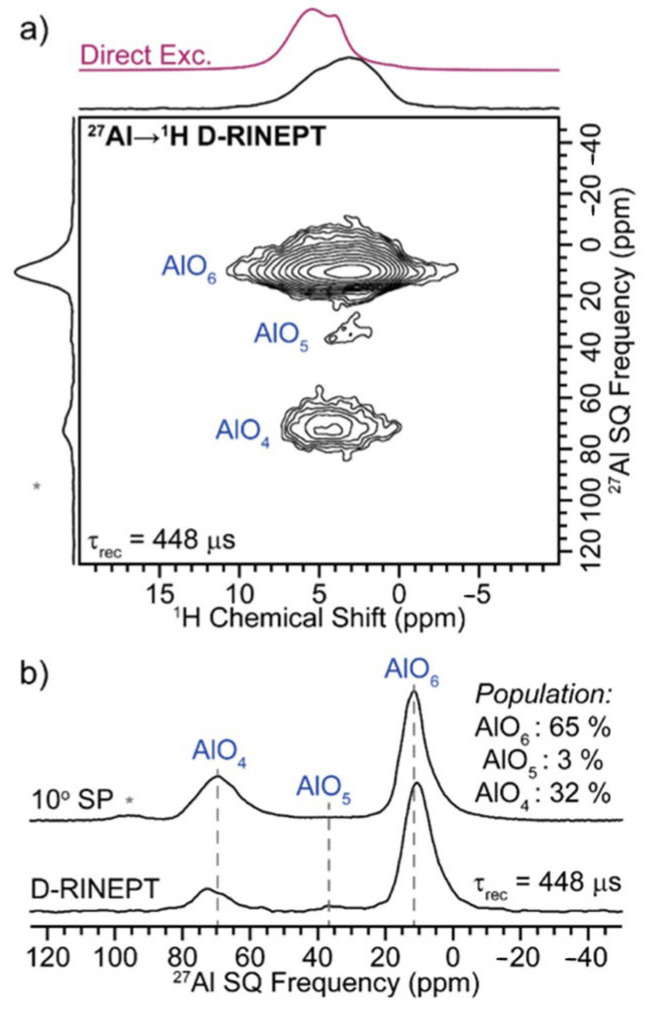
(**a**) 2D ^27^Al → ^1^H D-RINEPT NMR spectrum of the synthesized γ–Al_2_O_3_ nanocrystals recorded at *B*_0_ = 19.6 T with a 17.857 kHz MAS frequency and 448 μs of total SR412 dipolar recoupling applied to the ^1^H spins. The direct excitation ^1^H NMR spectrum is shown above the 2D ^1^H projection. (**b**) Comparison of a quantitative (upper) 10° tip-angle single-pulse (SP) (*) ^27^Al NMR spectrum with that of the (lower) 2D ^27^Al → ^1^H D-RINEPT ^27^Al projection.

We further investigated the local structure of the plasma synthesized γ-Al_2_O_3_ nanocrystals via high-field (19.6 T) ^1^H and ^27^Al magic-angle spinning (MAS) solid-state NMR spectroscopy. A two-dimensional (2D) ^27^Al → ^1^H dipolar-refocused insensitive nuclei enhanced by the polarization transfer (D-RINEPT) NMR spectrum reveals a broad ^1^H NMR signal centered at ca. 3 ppm, correlating to three ^27^Al NMR signals centered at ca. 12, 35 and 70 ppm, which are assigned to AlO_6_, AlO_5_ and AlO_4_ species, respectively (Figure 3a). The short duration of dipolar recoupling (τ_rec_ = 448 μs) ensures that the observed Al sites are likely on the surface of the nanocrystals and capped with hydroxyl groups. A ^1^H dipolar double-quantum-single-quantum (DQ-SQ) homonuclear correlation NMR spectrum reveals that the hydroxyl groups are clustered (i.e., spatially proximate to each other) on the surface of the nanocrystal, consistent with the broad stretching band observed in the FT-IR spectrum (Figure S1). Integration of a quantitative 10° single-pulse (SP) ^27^Al NMR spectrum reveals that the population of AlO_6_, AlO_5_ and AlO_4_ is ca. 65, 3 and 32%, respectively (Figure 3b). The much larger population of AlO_4_ observed in the SP ^27^Al NMR spectrum, as compared to the surface-selective D-RINEPT NMR spectrum, suggests that AlO_6_ predominantly terminates the surface of the nanocrystal (Figure 3b). Lastly, we recorded 2D ^27^Al dipolar DQ-SQ NMR spectra to probe the AlO_x_-AlO_x_ (x = 4–6) linkages with the nanocrystals (Figure S2). The 2D ^27^Al DQ-SQ NMR spectra reveal intense AlO_6_-AlO_6_ and AlO_6_-AlO_4_ homonuclear correlations, suggesting that the majority of the nanocrystal contains AlO_6_ linked to either another AlO_6_ or AlO_4_, consistent with prior NMR experiments on γ-Al_2_O_3_ [48]. There were additional weak AlO_4_-AlO_4_ correlations, suggesting that this linkage is relatively rare. No correlations involving AlO_5_ were observed due to the low population of the AlO_5_ site. We note that the observed ^27^Al homonuclear correlations of the synthesized γ-Al_2_O_3_ nanocrystals are near identical to that of commercially available γ-Al_2_O_3_ (Figure S2).

**Figure 4:**
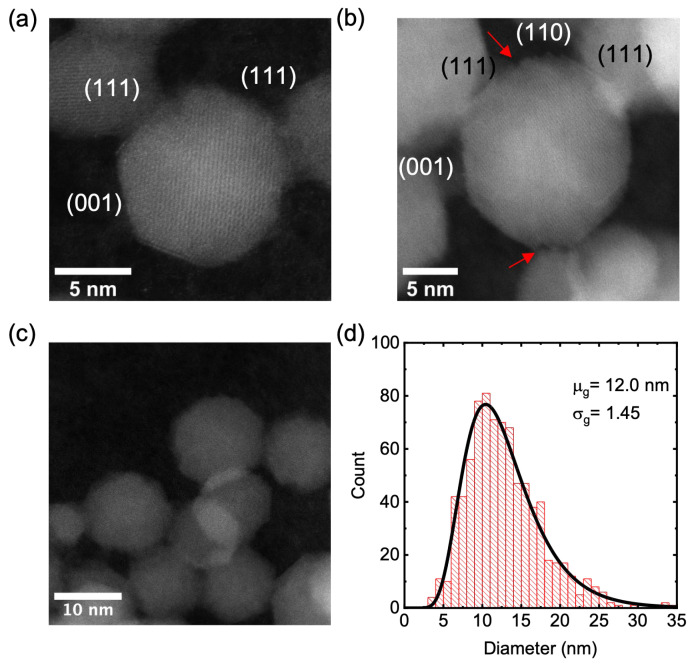
HAADF-STEM analysis of γ-alumina NCs. (**a**) Average-sized (~11 nm) (110)-oriented NC showing (111) and (001) facets. The outer atomic layer of (111) facets exhibit enhanced contrast due to excess Al^3+^ cations. (**b**) Larger (~16 nm) (110)-oriented NC showing (111) and (001) facets. The (110) facets have a stepped structure, exposing alternating (111) facets (red arrows). (**c**) Representative image of a collection of NCs. (**d**) Size distribution of 800 NCs. The mean μ_g_ and geometric standard deviations σ_g_ were estimated by fitting the histogram with a log-normal distribution.

HAADF-STEM images were analysed to examine the morphology and the size distribution of plasma synthesized γ-Al_2_O_3_ nanocrystals. Figure 4 shows representative HAADF-STEM images and the size histogram of γ-Al_2_O_3_ nanocrystals. Overall, the sample consisted of well-dispersed, facetted nanocrystals with an average crystal diameter of 12.0 nm with a geometric standard deviation of 1.45. The primary morphology of these particles appeared to be cuboctahedral [32], exposing (111), (110), and (001) facets. High-resolution images revealed that the (110) surface was not atomically flat but consisted of stepped facets of alternating (111) surfaces, as shown in Figure 4b.

**Figure 5:**
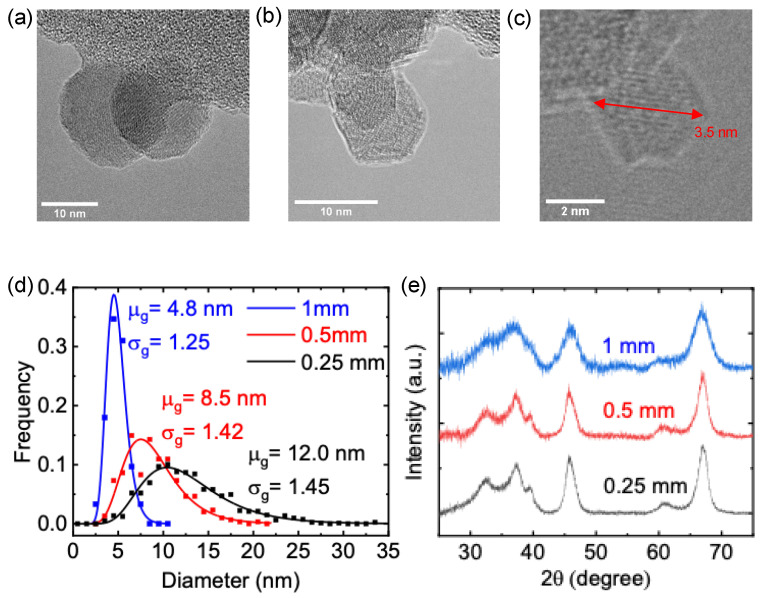
TEM images of γ-Al_2_O_3_ nanocrystals (**a**) with 0.25 mm orifice width at 3.8 Torr pressure, (**b**) with 0.5 mm orifice width at 2.5 Torr pressure, (**c**) with 1 mm orifice width at 1.3 Torr pressure (red arrow indicates the shown particle dimension), (**d**) respective particle size distributions, and (**e**) XRD patterns.

This observation of surface reconstruction of the (110) surface is consistent with the DFT calculations by Pinto et al., suggesting that the (110) facet was thermodynamically favored to reconstruct into (111) facets [67]. Furthermore, enhanced surface contrast was observed in (111) terminating facets (Figure 4a and Figure 5a,b), which corroborates previous literature reports of facetted γ-Al_2_O_3_ nanocrystals [7,31,32]. The enhanced surface contrast found in cuboctahedral γ-Al_2_O_3_ nanocrystals is hypothesized [31] to occur due to the excess Al^3+^ cations on (111) surface planes. The presence of excess Al^3+^ cations at the nanoparticle’s surface is a modification from the bulk structure, which is found to be critical in improving the dispersion and the thermal stability of fine metal particle catalysts in γ-Al_2_O_3_ catalyst supports [31].

To evaluate the impact of total reactor pressure on the size of γ-Al_2_O_3_ nanocrystals, we varied the reactor pressure by tuning the orifice diameter at constant volumetric flow rates. The orifice width was tuned to 0.25 mm, 0.5 mm, and 1 mm, resulting in 3.8 Torr, 2.5 Torr, and 1.3 Torr total reactor pressures, respectively. The size of γ-Al_2_O_3_ nanocrystals decreased with decreasing reactor pressure, producing 12.0 nm particles at 3.8 Torr, 8.5 nm particles at 2.5 Torr, and 4.8 nm particles at 1.3 Torr with geometric standard deviations of 1.45, 1.42 and 1.25, respectively (Figure 5a–d). The morphology of γ-Al_2_O_3_ nanocrystals remained similar under different pressure conditions exhibiting their typical facetted nature. In many studies, it has been found that the average nanoparticle size correlates nearly linearly with the residence time of particles in the plasma [68]. A slower gas velocity yields longer residence time, thus increasing the size of particles leaving the plasma. Here, for essentially the same total of volumetric flow rate, a wider orifice reduces the pressure in the reactor while increasing the gas velocity. Therefore, an increasing orifice size results in a decreasing residence time and a reduction of the average particle sizes of γ-Al_2_O_3_ nanocrystals. Assuming 2 cm for the plasma length, the gas residence times are estimated as 33 ms, 22 ms, and 10 ms with the orifice width of 0.25 mm, 0.5 mm, and 1 mm, respectively. Here, the average nanoparticle diameters are found to increase nearly linearly with the estimated gas residence times. Recently, we found the size of the particles leaving a flow-through nonthermal capacitively coupled plasma reactor is mainly determined by the balance of gas drag forces and electrostatic forces acting on the particles [69]. However, it is unclear whether trapping plays a role in the inductively coupled plasma reactor.

XRD patterns confirmed the crystalline nature of γ-Al_2_O_3_ nanoparticles synthesized under all three pressure conditions (Figure 5e). At the nanoscale, the thermodynamics of the growth process drives the crystalline structure of Al_2_O_3_ nanocrystals. It was reported that Al_2_O_3_ nanoparticles larger than ~20 nm should adopt an α structure, while Al_2_O_3_ nanoparticles smaller than ~6.5 nm will be amorphous [70]. Hence, γ-Al_2_O_3_ nanocrystals were predicted to be thermodynamically stable in the range ~20–6.5 nm [70]. Our approach demonstrated that the size tuning of γ-Al_2_O_3_ is feasible in a range of 12–5 nm with some nanocrystals as small as 3.5 nm, as shown in Figure 5c, which is below the thermodynamically predicted size limit. It will be interesting to explore whether the nonthermal plasma environment, where nanoparticles are charged while they grow, more generally allows for the synthesis of material phases outside of thermodynamically predicted size limits.

## 4. Conclusions

We demonstrated the synthesis of γ-Al_2_O_3_ nanocrystals using an inductively coupled nonthermal plasma. While XRD patterns confirmed phase-pure crystalline γ-Al_2_O_3_ nanoparticles, TEM images revealed cuboctahedra morphology with (111) stepped facets. The total pressure of the reactor was varied by tuning the orifice diameter at a constant gas flow to yield γ-Al_2_O_3_ particles ranging between 5–12 nm. We observed γ-Al_2_O_3_ nanocrystals as small as 3.5 nm, which is below the size at which thermodynamics would predict amorphous alumina to be the most stable phase.

Overall, this study demonstrates the single-step synthesis of size-tunable, facetted γ-Al_2_O_3_ nanocrystals without additional post-synthetic calcination or annealing steps. These particles, specifically the γ-Al_2_O_3_ as small as 3.5 nm_,_ could be used as heterogeneous catalysts and catalytic supports. Their specific surface areas along with catalytic performance need to be probed in future work.

## Data Availability

The data that support the findings of this study are available from the corresponding authors upon reasonable request.

## Supplementary Materials

The following supporting information can be downloaded at: https://www.mdpi.com/article/10.3390/nano13101627/s1, Figure S1: 2D 1H dipolar DQ-SQ homonuclear correlation NMR spectrum recorded with a 17.857 kHz MAS frequency and 112 μs (i.e., two rotor cycles) of total homonuclear dipolar recoupling; Figure S2: 2D 27Al dipolar DQ-SQ homonuclear correlation NMR spectra of plasma synthesized gamma alumina nanocrystals and commercially available gamma alumina.
