# Supplementary material for: Inductively Coupled Nonthermal Plasma Synthesis of Size-Controlled γ-Al2O3 Nanocrystals
Source: Nanomaterials (Basel). 2023 May 12;13(10):1627. doi: 10.3390/nano13101627 (PMC10221976; doi:10.3390/nano13101627)
Supplement: Supplementary file 1 [file nanomaterials-13-01627-s001.zip › nanomaterials-2363610-supplementary.pdf]

Inductively Coupled Nonthermal Plasma Synthesis of Size-Controlled γ-Al_2O_3 Nanocrystals

Zichang Xiong [1,†], Himashi P. Andaraarachchi [1,†], Jacob T. Held [2], Rick W. Dorn [3], Yong-Jin Jeong [1], Aaron Rossini [3] and Uwe R. Kortshagen [1,*]

1 Department of Mechanical Engineering, University of Minnesota, 111 Church Street SE, Minneapolis, MN 55455, USA; xion1832@umn.edu (Z.X.); handaraa@umn.edu (H.P.A.); yjjeong@ut.ac.kr (Y.-J.J.)
2 Chemical Engineering and Materials Science Department, University of Minnesota, Minneapolis, MN 55455, USA; jaheld@ethz.ch
3 Ames National Laboratory, United States Department of Energy, Department of Chemistry, Iowa State University, Ames, IA 50011, USA; rwdorn@iastate.edu (R.W.D.); arossini@iastate.edu (A.R.)
* Correspondence: kortshagen@umn.edu
† These authors contributed equally to this work.

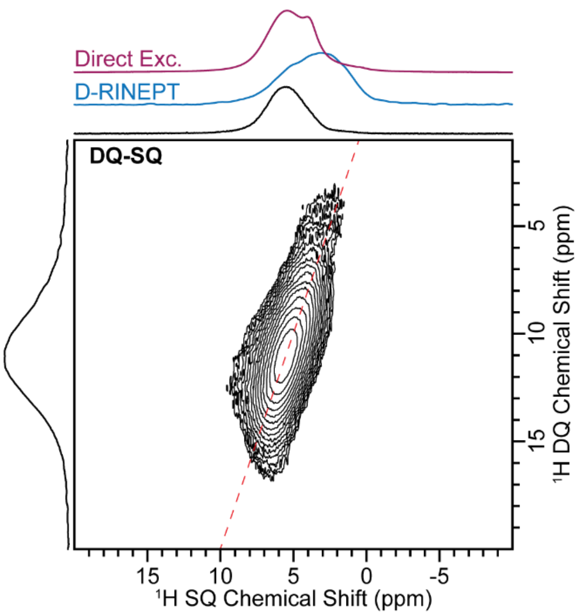

Figure S1. 2D ^{1}H dipolar DQ-SQ homonuclear correlation NMR spectrum recorded with a 17.857 kHz MAS frequency and 112 μs (i.e., two rotor cycles) of total homonuclear dipolar recoupling. Direct excitation ^{1}H and $^{27}Al\rightarrow{}^{1}H$ D-RINEPT NMR spectra are overlaid above the ^{1}H SQ projection.

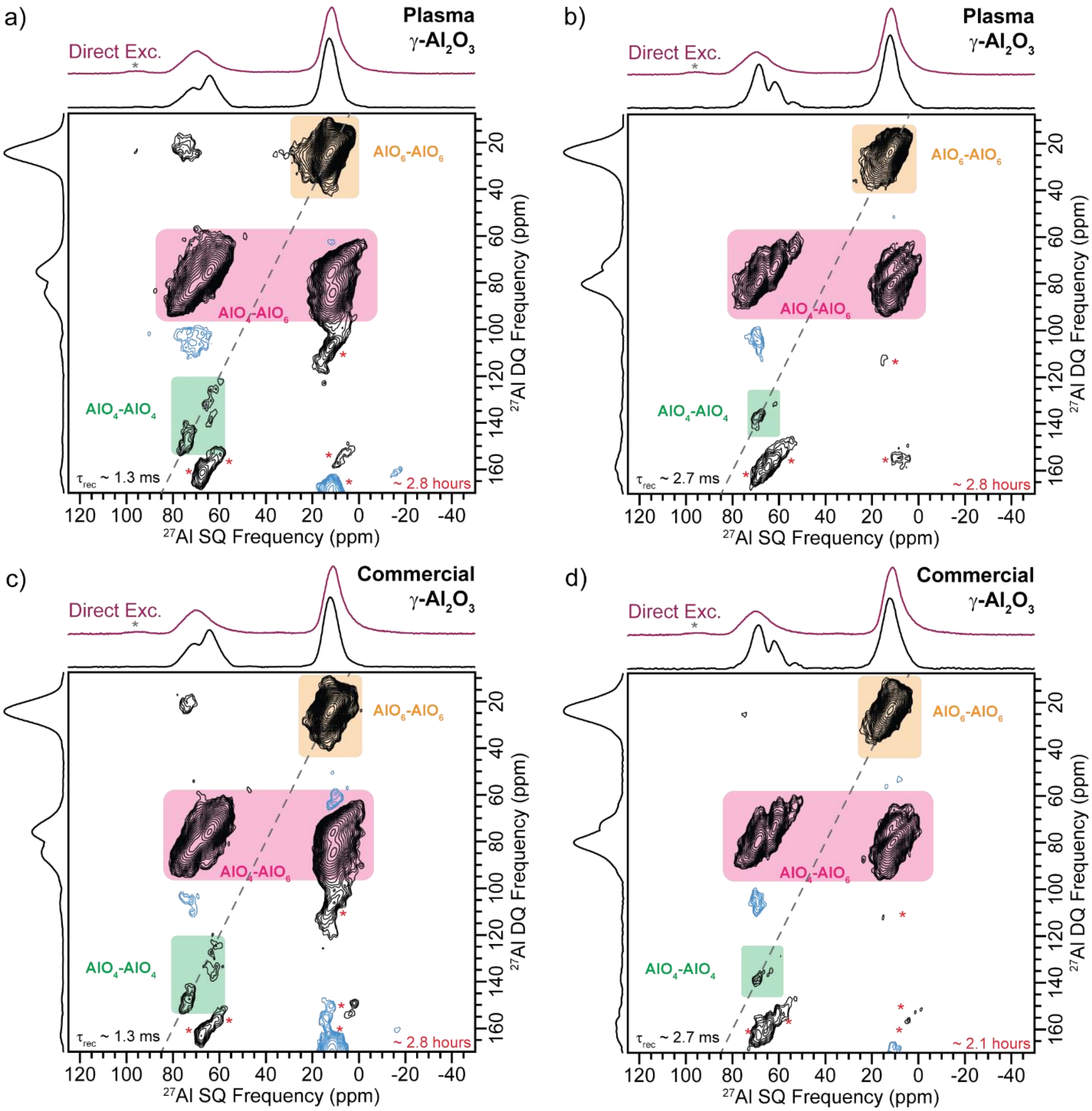

Figure S2. 2D ^{27}Al dipolar DQ-SQ homonuclear correlation NMR spectra of (a-b) plasma synthesized gamma-alumina nanocrystals and (c-d) commercially available gamma-alumina recorded at B_0 = 19.52 T with a 17.857 kHz MAS frequency and either (a, c) 1.3 ms or (b, d) 2.7 ms of total $BR2_2^1$ homonuclear dipolar recoupling. The asterisk (*) correspond to spinning sidebands.
